# ESSKA and EBJIS recommendations for the management of infections after anterior cruciate ligament reconstruction (ACL-R): prevention, surgical treatment and rehabilitation

**DOI:** 10.1007/s00167-023-07463-3

**Published:** 2023-05-27

**Authors:** Daniel Pérez-Prieto, Trifon Totlis, Tomislav Madjarevic, Roland Becker, Christen Ravn, Juan C. Monllau, Nora Renz

**Affiliations:** 1 Department of Traumatology and Orthopaedic Surgery, Hospital del Mar, Barcelona, Spain; 2 IcatKNEE, Hospital Universitari Dexeus – Universitat Autònoma de Barcelona (UAB), Barcelona, Spain; 3 Thessaloniki Minimally Invasive Surgery (The-MIS) Orthopaedic Centre, St. Luke’s Hospital, Thessaloniki, Greece; 4 School of Medicine, Faculty of Health Sciences, Aristotle University of Thessaloniki, Thessaloniki, Greece; 5 University Hospital for Orthopaedic Surgery Lovran, Faculty of Medicine, University of Rijeka, Rijeka, Croatia; 6 Centre of Orthopaedics and Traumatology, University of Brandenburg Theodor Fontane, Brandenburg, Germany; 7 Department of Orthopaedic Surgery and Traumatology, Aarhus University Hospital, Aarhus, Denmark; 8 Center for Musculoskeletal Surgery (CMSC), Charité - Universitätsmedizin Berlin, corporate member of Freie Universität Berlin, Humboldt-Universität zu Berlin, and Berlin Institute of Health, Berlin, Germany; 9 Department of Infectious Diseases, Bern University Hospital, University of Bern, Bern, Switzerland

**Keywords:** Infection after anterior cruciate ligament reconstruction, Prevention, Management, Arthroscopy, Debridement, Reoperation

## Abstract

**Purpose:**

Infection after anterior cruciate ligament reconstruction (ACL-R) is a rare but severe complication. Despite an increase in articles published on this topic over the last decade, solid data to optimized diagnostic and therapeutic measures are scarce. For this reason, the European Bone and Joint Infection Society (EBJIS) and the European Society for Sports Traumatology, Knee Surgery and Arthroscopy (ESSKA) collaborated in order to develop recommendations for the diagnosis and management of infections after ACL-R. The aim of the workgroup was to perform a review of the literature and provide practical guidance to healthcare professionals involved in the management of infections after ACL-R.

**Methods:**

An international workgroup was recruited to provide recommendations for predefined clinical dilemmas regarding the management of infections after ACL-R. MEDLINE, EMBASE, Cochrane Library and Scopus databases were searched for evidence to support the recommended answers to each dilemma.

**Results:**

The recommendations were divided into two articles. The first covers etiology, prevention, diagnosis and antimicrobial treatment of septic arthritis following ACL-R and is primarily aimed at infectious disease specialists. This article includes the second part of the recommendations and covers prevention of infections after ACL-R, surgical treatment of septic arthritis following ACL-R and subsequent postoperative rehabilitation. It is aimed not only at orthopedic surgeons, but at all healthcare professionals dealing with patients suffering from infections after ACL-R.

**Conclusion:**

These recommendations guide clinicians in achieving timely and accurate diagnosis as well as providing optimal management, both of which are paramount to prevent loss of function and other devastating sequelae of infection in the knee joint.

**Level of evidence:**

V.

## Introduction

Anterior cruciate ligament (ACL) injury is common, especially in young and active patients. The gold standard for restoring joint stability and allowing return to competitive pivoting sports is ACL reconstruction (ACL-R). Infection after ACL-R is a rare but severe complication, which can significantly affect the short and long-term postoperative clinical outcomes [11, 55]. In cases of delayed diagnosis and suboptimal therapy, infection may result in the degeneration of cartilage in the knee joint [20].

Recommendations for the management of septic arthritis after ACL reconstruction were reported in the systematic review by Wang et al., including studies up to 2012 [70]. However, the increase in articles published in this field in the last decade has refocused clinicians and researchers on the topic [2, 6, 17]. Nevertheless, solid data on optimized diagnostic and therapeutic approaches are scarce. The lack of updated comprehensive guidelines underlines the necessity to summarize the current literature and provide practical recommendations for the management of infections after ACL-R. For this reason, the European Bone and Joint Infection Society (EBJIS) and the European Society for Sports Traumatology, Knee Surgery and Arthroscopy (ESSKA) collaborated in order to develop recommendations for the diagnosis and management of infections after ACL-R.

The aim of the workgroup was to perform a review of the literature and provide a practical guidance for healthcare professionals involved in the management of infections after ACL-R.

## Materials and methods

A steering committee identified 28 clinical dilemmas related to infection after ACL-R and grouped these into five categories, namely: (a) etiology, risk factors and prevention; (b) diagnosis; (c) surgical treatment; (d) systemic antimicrobial treatment; and (e) rehabilitation. A workgroup of international experts was recruited among members of EBJIS and ESSKA to provide recommendations for each dilemma. The literature reviews were performed by members of the workgroups in order to identify the available evidence to support the recommendations for each dilemma. MEDLINE, EMBASE, Cochrane Library and Scopus databases were searched for articles regarding septic arthritis and ACL reconstruction and surgical treatment and rehabilitation. Only clinical studies written in English were included. Basic science studies, letters to the editor and case reports were excluded. Altogether 101 studies were identified. Most of the studies were Level IV or Level V studies. Due to the low incidence of infection after ACL-R and the resulting paucity of relevant high-quality studies, many recommendations or suggestions were extrapolated from other joint-related infections such as septic arthritis or periprosthetic joint infections, for which more high-quality studies are available.

The recommendations were divided into two articles. The first covers etiology, prevention, diagnosis and antimicrobial treatment of septic arthritis following ACL-R and is primarily aimed at infectious disease specialists [51]. This article includes the second part of the recommendations covering prevention of infections after ACL-R, surgical treatment of septic arthritis following ACL-R and subsequent post-operative rehabilitation. A summary of the recommendations regarding diagnosis of infections after ACL-R is also included. This study is aimed not only at orthopedic surgeons, but also at all healthcare professionals dealing with patients suffering from infections after ACL-R. Part of these recommendations was previously published within the guidelines for management of septic arthritis in native joints [50].

## Prevention

Risk factors for infection after ACL-R include previous corticosteroid injection, concomitant surgical procedures and young age [9, 30, 59]. A 3-day skin and nasal universal decolonization prior to ACL-R is suggested in all hospitals in which it can be implemented, taking into account that this recommendation may not be cost-effective [24, 72]. For intravenous antibiotic prophylaxis, one single dose of a cephalosporin 30–60 min before ACL-R is recommended. In cases of type 1 allergy to penicillins or cephalosporins, prior knee infection, antibiotic therapy or known MRSA carriage prophylaxis with one dose of 1 g of vancomycin (or 15 mg/kg, max. 2500 mg) can be used. Postoperative antibiotic (either intravenous or oral) is unnecessary [30, 71].

1. **What is the role of preoperative knee aspiration with regard to development of infections after ACL-R?**

In cases of native joints, the risk of infection is very low (< 0.01% after arthrocentesis), although it is supposedly higher in cases of knee hemarthrosis after injury due to the inflammatory response [16]. Therefore, aspiration is indicated only in cases of limited range-of-motion (ROM), the inability to activate quadriceps muscle or unbearable pain caused by hemarthrosis. Corticosteroids have been described as risk factors for infection after ACL-R in the first part of the ESSKA and EBJIS recommendations and should be avoided in patients scheduled for ACL-R. There is no data regarding the optimal timing to safely perform ACL reconstruction following a corticosteroid injection. According to the systematic review by Baums et al., the risk of periprosthetic joint infection is not increased when total knee arthroplasty is postponed for 6 months after the last corticosteroid injection [7]. Based on the latter study and the recommendations of Conen et al. for native joints [15, 16], we would recommend postponing surgery (if possible) for 6 months if the patient received a corticosteroid injection before injury. However, relevant evidence is needed to support this statement. The indication for ACL surgery will primarily depend on degree of instability, symptoms and the risk of developing concomitant pathologies.

***Evacuating post-injury hemarthrosis is recommended only in cases of uncontrolled pain and limited ROM. Corticosteroid injection should be avoided for at least 6 months before ACL-R.***

2. **Is there a preferred graft type to minimize infection after ACL-R?**

Graft choice is a controversial issue in the field of ACL-R. With regard to autografts, studies have found a significantly higher risk of infection when hamstrings were used compared to bone-patellar tendon-bone (BPTB) graft [13, 21, 34, 37]. A recent meta-analysis confirms this finding [3]. When hamstrings are compared to quadriceps tendon autograft, the latter may result in a significantly lower risk of infection as shown in one retrospectively designed study on revision ACL-R [56]. Several studies that implement new preventive strategies such as the vancomycin soaking technique (see point 3) have reported similar infection rates of close to 0% either in hamstrings or BPTB autografts [46]. These studies were not aimed at comparing the infection rates between BPTB and hamstrings [37]. In general, it is recommended that the choice of graft should be in accordance with the surgeons’ experience and patients’ characteristics rather than taking infection risk as a basis for decision-making when the vancomycin technique is used. Intuitively, allografts are not at greater risk of infection [4]. Although there are no randomized controlled trials, several studies confirm this, including a recent meta-analysis [3]. Thus, the use of either an autograft or allograft, based on patient characteristics rather than infection risk, is recommended [22].

***Allografts and autografts have a similar infection rate. Therefore, choosing the graft based on surgeons’ experience and patients’ characteristics is recommended. A hamstring autograft seems to have a greater risk of infection after ACL-R compared to BPTB. However, the surgeon may use either graft depending on the patients’ characteristics when the vancomycin technique is used.***

3. **What is the evidence and the recommendation for ACL graft soaking in a vancomycin solution?**

The technique of soaking the ACL graft in a vancomycin solution was first described by Vertullo et al. in 2012. A 5 mg/ml vancomycin solution is used to soak the graft for 10–15 min in order to eradicate contamination and also provide an antibiotic reservoir [23, 29, 45, 68]. A close to 0% infection rate has been found in all relevant studies, in both hamstring or BPTB autograft [5, 18, 42, 46, 47]. Two recent meta-analyses that included 3,000 and 8,700 ACL-R using the vancomycin technique have also obtained similar results with an incidence of 0% and 0.01% infections after ACL-R, respectively, when this technique was used [19, 41]. Additionally, recent studies have proven it to be a safe technique with no increase in re-ruptures and a similar return-to-play time [10, 26, 42]. Moreover, it is a cost-effective technique [32, 42]. However, it is important to remember that all other measures to reduce infection including behavior in the operating room must be implemented and cannot be neglected. All the cited studies include primary reconstructions without other procedures, in which infection risk is higher. Soaking techniques using other antibiotics, e.g., gentamicin, have also been described [74]. However, evidence is more limited and the activity against staphylococci is inferior to vancomycin. The resistance of microorganisms to vancomycin is very rare. It has mostly been described in enterococci, which is an infrequent pathogen in infections after ACL-R.

***Soaking the ACL graft in a 5 mg/ml solution of vancomycin is recommended as the technique reduces infection after ACL-R.***

## Diagnosis

Diagnosis of infection after ACL-R is based on patient history, physical examination, laboratory parameters suggestive of an infectious process and analyses of synovial fluid following joint aspiration. The signs and symptoms suggestive of infection include delayed ROM recovery, hyperthermia or swelling, wound drainage and arthrofibrosis as well as unusual pain and systemic symptoms such as fever and malaise [65, 70]. When skin redness, increased temperature and tenderness or delayed wound healing are located at the site where the graft was harvested, then a superficial infection at the harvest site is included in the differential diagnosis. Although superficial infections may occur, due to the proximity between graft harvest site and tibial tunnel, anteromedial portal and intra-articular cavity, the investigation for septic arthritis should be completed in all cases.

CRP as a systemic inflammatory parameter shows high sensitivity in acute infection conditions and low sensitivity in chronic infections. A secondary increase in CRP in the postoperative course is suggestive of infection as is a tenfold elevation of the normal value in the first postoperative week [36, 52]. The value of imaging in infections after ACL-R is secondary. Magnetic resonance imaging (MRI) provides information about soft tissue, such as synovitis or cyst formation. It may be used in doubtful cases to exclude other causes of an unfavorable postoperative course and complications.

Aspiration is mandatory in cases of suspicious of infection after ACL-R. It needs to be performed prior to administration of antibiotics. Antibiotic pretreatment may affect the microbiological examination and should be withheld until synovial fluid has been harvested. The determination of the leukocyte count and percentage of polymorphonuclear cells in synovial fluid is the cornerstone in the diagnosis of infections after ACL-R. A normal leukocyte count (< 2.000 /µl) rules out infection in most cases. A granulocytes percentage of > 90% shows a high likelihood ratio for infection [40]. Microbiological cultures of synovial fluid should preferably be inoculated in pediatric blood culture bottles and plated on solid media. In cases of acute infection at a later stage after ACL-R, crystal arthropathy should be excluded through microscopy. In the case of an acute onset of local and systemic signs and symptoms of knee infection at any time after an uneventful period after ACL-R, blood cultures should be collected to exclude hematogenous infection.

During arthroscopic debridement for septic arthritis after ACL-R, the surgeon is recommended to collect and analyze synovial fluid for microbiological analysis (blood cultures bottles and native vials), five tissue samples from representative and macroscopically infected tissue and at least one sample for histopathological examination. In cases of removal or exchange of the graft and/or the fixation devices, the graft should be sent for microbiological culture and foreign material (fixation devices) to sonication. The laboratory should be notified about the type of infection and that a foreign body is involved in the infection to ensure prolonged incubation of the samples. Confirmative criteria for infection after ACL-R are intraarticular purulence, purulent secretion or sinus tract communication with the joint, positive cultures of tissue, synovial fluid or sonication and/or histopathology consistent with acute infection [64, 65, 73].

## Surgical treatment

4. **Surgical treatment is necessary for an infection after ACL-R. Which type of surgery is recommended?**

The key aims in the management of infection after ACL-R are successful infection clearance and the complete functional recovery of the knee joint [27, 28, 67]. The primary endpoint of previous studies comparing conservative treatment and surgical debridement was infection cure without any assessment of functional outcomes or cartilage damage [35, 49].

Despite the lack of evidence, the present group recommends arthroscopic debridement for the following reasons:

- Surgical treatment is necessary to wash out proteolytic enzymes and toxins which cause chondrocyte degeneration [54, 58].
- Prompt evacuation of the joint by means of arthroscopic revision reduces the bacterial load and intraarticular pressure [64].
- Arthroscopy allows for cartilage evaluation in accordance with the Gächter classification and the evaluation of graft stability and viability [61, 62].
- Compared to open surgery, arthroscopy has proven to be less invasive and facilitates a faster recovery, without compromising the cure rate [44].

However, open debridement is indicated in cases when there is subchondral bone involvement (Gächter IV) [62].

Although there are old reports of successful treatment with percutaneous drainage or bedside arthrocentesis [35, 49], the current literature recommends arthroscopic debridement and intravenous antibiotic therapy as the treatment of choice for septic arthritis after ACL-R [31, 43].

***Arthroscopic debridement in combination with antibiotic therapy is recommended as the primary therapeutic option in every patient. In the rare case of inoperability, repeated needle aspiration might be an alternative.***

5. **What is the best time for surgical treatment?**

Intraarticular infection can lead to graft failure, joint stiffness and chondral damage, and therefore arthroscopic surgery is required as soon as possible [14]. Delaying surgical treatment leads to chondrocyte degradation and ACL graft insufficiency, which can cause functional disability. Therefore, surgical treatment should be performed immediately when infection is considered. Delayed treatment and prolonged intra-articular infection have been shown to compromise graft function [56]. The cartilage loses more than half of its glycosaminoglycan and collagen if surgical treatment is not initiated within 7 days of the onset of symptoms [58, 60]. Delayed treatment of more than 7 days, late presentation (more than 30 days after surgery) and virulent microorganisms are risk factors for prolonged treatment and the need for an arthrotomy and graft and hardware removal [25, 27, 48, 53, 69]. Dave et al. reported a correlation between the number of hours between the onset of symptoms and index surgery and the need for multiple procedures [28].

***Surgical treatment is recommended as soon as a clinical suspicion is established in cases with acute symptoms or in the early postoperative period, even if the microbiological results are still pending.***

6. **How many arthroscopic procedures should be performed?**

Wang et al. reported that 60% of the patients were successfully treated with a single arthroscopic debridement. Repeated debridement was carried out because of persistent clinical symptoms, fever or increased CRP levels [69]. Additional arthroscopic debridement is indicated in cases of persistent septic arthritis, persistent microorganism in cultures or clinical symptoms (i.e., local inflammatory changes, persistent wound drainage, fever) and pathologic laboratory results (i.e., persistent or secondarily increased CRP) [14, 27]. Binnet et al. showed that an average of 2.66 procedures was required to eradicate infection [8]. In previous reports, the graft along with its original fixation material remained in place when the graft was considered functional and did not block knee motion [39]. Indeed, several reports have shown that about four out of five ACL grafts can be successfully salvaged with multiple debridement procedures. However, Vertullo et al. suggest that failure to see an improvement after two arthroscopic irrigations implies that the bacteria have formed a biofilm, and the graft is non-viable or that osteomyelitis has also involved the femoral tunnel [68]. Calvo et al. repeated joint lavage several times [14]. After the third lavage, the graft and implants were removed because of a persistent clinical infection, macroscopic graft damage or elevated inflammation parameters. However, those patients had presented more than one week after infection started. In cases with persistent infection, MRI should be considered to evaluate the possible involvement of the tunnels and to detect cavities or abscess formation [48]. McAllister et al. were able to retain the graft by managing persistent infections with two to four subsequent debridement procedures, but all four patients developed degenerative changes at a mean of 36 months (range, 28 to 42 months) [39]. One factor that is crucial for graft viability is an early diagnosis, since patients diagnosed after 7 days from the onset of symptoms have a higher graft removal rate [53, 69]. Delayed treatment may weaken the graft, delay integration or lead to insufficiency [56].

Scheduled debridement should not be performed in patients with a favorable course after the first debridement.

***Additional debridement is indicated if the clinical course is not favorable. Unfavorable determinants include increasing pain, fever or persistent or secondarily increased CRP without any other explanation (e.g., nosocomial infection), a persistent drainage from the portal or persistent local signs of inflammation. In cases with an uneventful course, repeated arthroscopic debridement is not needed. If the course is not adequate after the third debridement, graft and hardware removal should be considered. In cases with persistent infection, MRI may help to evaluate bony involvement.***

7. **What is the role of the Gächter classification in infections after ACL-R?**

Intraoperative arthroscopic findings in native septic arthritis were described and stratified by Gächter et al. and are shown in Table 1 [62]. This classification is a useful guide to the therapeutic measures to take. It recommends mild arthroscopic debridement of necrotic tissue and adhesiolysis only using the shaver or radiofrequency, without synovectomy in stage I (mainly lavage) because the synovial membrane acts as a barrier and provides the joint with nutrients and antibiotics. Moreover, complete removal, when it is not affected by infection, will provoke bleeding and hematoma. Aggressive arthroscopic debridement is recommended in stages II and III and open debridement (arthrotomy) in stage IV. This classification also predicts the need for further debridement, 52% in stage II and 75% in stage III [62].

**Table 1:** Clinical staging of native septic arthritis by Gächter et al. [55]

Clinical staging (Gächter classification)	Intraoperative (arthroscopic) spread of inflammatory process
Stage 1	Turbid effusion and hyperemic synovia
Stage 2	Purulent effusion, fibrinous appositions and hypertrophic synovia
Stage 3	Synovial adhesion, necrotic areas of synovia and cartilage
Stage 4	Cartilage necrosis, bone erosion and osteolysis

Although several authors recommend choosing the surgical strategy according to the Gächter staging, there is no study that has validated its utility in infections after ACL-R. Nevertheless, the use of the Gächter classification is recommended as a surgical and prognostic guide in infections after ACL-R. It should be considered in combination with graft viability and hardware stability.

***The Gächter classification along with graft condition is recommended as a guide to decide whether to perform arthroscopic debridement with graft retention or debridement with graft and hardware removal for ACL-R infections.***

8. **In which situations should the graft and hardware be removed? When can the new ACL-R be performed after graft and hardware removal?**

Graft removal should be considered when multiple debridement procedures fail to control the infection, and fixation devices are loose or in cases with graft insufficiency [28]. Further, graft and hardware removal should be performed in cases of bony involvement of the tibia or femur (Gächter IV) [56].

According to several studies, revision ACL-R may be considered three to six months following graft removal [3, 28, 40, 60]. However, this approach was recommended in patients with additional osteomyelitis rather than in primary infection after ACL-R.

Based on the available evidence on PJI and native septic arthritis, a new graft can be reconstructed in a shorter period (6 weeks) if the following criteria are fulfilled [75]:

- No bone involvement (no osteomyelitis)
- Favorable clinical evolution
- Decreasing CRP (normal value is not required)
- Absence of difficult-to-treat infections caused by a microorganism that is resistant to biofilm-active antibiotics

At the time of repeat ACL-R, tissue cultures from the synovial membrane and bone tunnels must be obtained during surgery. Histopathology of the tunnels may be of help in ruling out osteomyelitis. Keeping patients on antibiotics until the results of intraoperative diagnostics are available is recommended.

If these requirements are not met, the new ACL-R must be delayed, either after additional debridement procedures or when the prolonged antibiotic treatment (i.e., osteomyelitis treatment) is completed.

***Graft and hardware must be removed in cases of multiple debridement procedure failures and/or hardware loosening/graft insufficiency. In selected cases ACL revision surgery can be performed 6 weeks after graft and hardware removal.***

Treatment of infections after ACL-R consists of a combination of surgical and antimicrobial therapy. Comprehensive presentation of antimicrobial treatment is included in the first part of recommendations, primarily addressing infectious disease specialists. In summary, immediately after joint aspiration empirical intravenous antibiotic therapy should be commenced in cases with strong clinical evidence of acute infection. The empirical treatment should start with a beta lactam/beta lactam inhibitor combination along with vancomycin or daptomycin until culture results are available. The recommended pathogen-specific treatment is analyzed in the first article. A 1-week (up to 2 weeks) course of intravenous treatment is suggested followed by oral treatment for another 4–5 weeks. Bactericidal agents with good bioavailability and bone penetration as well as biofilm activity in infections with any grafts and fixation devices in place are preferred. Good clinical response with nearly normal CRP values allows change to oral treatment.

## Rehabilitation

9. **Are functional outcomes impaired in patients after an ACL-R infection?**

A satisfactory result after treatment of an ACL-R infection should be defined as good functional scores, preserved articular cartilage architecture and ACL graft function, restoration of full ROM and a return to previous level of activity [64].

Some studies suggest that knee function is impaired after infection, and the results are inferior to those without an infection after ACL reconstruction [25, 53, 59]. Stiffness and arthrofibrosis are the most common reasons for limited function [33, 53]. Even after arthroscopic arthrolysis, a flexion or extension deficit may be seen in infected patients [25, 28, 39, 73]. Abdel-Aziz et al. found loss of ROM in 21% of the patients with an infection following ACL-R [1], whereas in the Judd et al. study, infected ACL-R did require a longer rehabilitation period compared to non-infected cases [28].

Conversely, Boström et al. reported no inferior objective knee function and patient satisfaction in 27 patients with infection after ACL-R in comparison with patients with an uncomplicated course after ACL-R, at a follow-up time of 60 months [11]. In a recent study comparing patients with initial graft retention and those with graft removal and consecutive revision ACL-R, there was no statistically significant difference concerning functional outcomes and return to sports rate [63]. Interestingly, in another study when the ACL-R infection went undetected for less than 5 days, no statistically significant differences were noted in the mean Lysholm, IKDC, or KOOS scores between the study groups [73]. It is well-known that delay in treatment could lead to graft failure, articular cartilage damage and joint dysfunction [48]. Prolonged septic arthritis can result in irreversible joint destruction and immediate treatment is imperative [12, 38].

***It is not clear whether functional scores are impaired in patients with infection after ACL-R. Early diagnosis and treatment are crucial to prevent arthrofibrosis, stiffness and cartilage destruction.***

10. **Which rehabilitation protocol is appropriate in cases of infection after ACL-R?**

Trueta and Barnes were the first to suggest immobilization of the limb when dealing with wound infection [66]. However, those studies were carried out 80 years ago and were mostly related to open fractures in war injuries. What is crucial to healing in a septic non-union might be harmful in terms of infections after ACL-R, because the main sequelae in infections after ACL-R is a decrease in ROM, as previously stated.

Physical therapy should focus on preventing stiffness and regaining ROM [27]. A loss of flexion, with a mean loss of 5.8°, was reported in 13 studies [28]. Thus, after arthroscopic irrigation and debridement, early mobilization and ROM exercises are recommended according to patient’s symptoms and pain [14]. The protocol also includes quadriceps and hamstrings strengthening through progressive isometric, isotonic and isokinetic exercises with close monitoring of symptoms [59, 64]. Weight-bearing may be allowed, as tolerated, from the early postoperative period [53, 57, 67].

***In patients with an infection after ACL-R, the same rehabilitation protocols as for primary ACL-R are recommended in order to prevent arthrofibrosis. Physical therapy is important for muscle strengthening and ROM improvement. However, the program should be adjusted according to patient’s symptoms and pain.***

## Abbreviations

ACL: Anterior cruciate ligament
ACL-R: Anterior cruciate ligament reconstruction
EBJIS: European bone and joint infection society
ESSKA: European society for sports traumatology, knee surgery and arthroscopy
ROM: Range of motion
BPTB: Bone-patellar tendon-bone
MRI: Magnetic resonance imaging
CRP: C-reactive protein
